# Potential Use of Gamma-Irradiated Ethnic Ready-to-Eat Foods to Improve the Nutritional Status of Landslide Victims

**DOI:** 10.3390/foods5030053

**Published:** 2016-07-26

**Authors:** Zubaidah Irawati Koenari, Carmen M. Siagian, Bona Simanungkalit, Asti Nilatany, Indra Mustika Pratama, Deudeu Lasmawati, Cecep M. Nurcahya

**Affiliations:** 1 Centre for Isotopes and Radiation Application (CIRA), National Nuclear Energy Agency, Jakarta 12440, Indonesia; asti_nita@yahoo.com (A.N.); a_indra_sheva@yahoo.com (I.M.P.); ndeu_1984@yahoo.co.id (D.L.); cecepnurcahya28@gmail.com (C.M.N.); 2 Indonesia Christian University, Community Medicine, Jl. LetjenSutoyo, Cawang, East Jakarta 13630, Indonesia; carsiag209@yahoo.com; 3 Centre of Applied Technology for Health and Clinical Epidemiology, Ministry of Health, Jl. Dr. Semeru, Bogor 16125, Indonesia; bonasimanungkalit@gmail.com

**Keywords:** cost benefits, ethnic ready to eat foods, ionizing radiation, landslide victims, nutritional status

## Abstract

The safety and high quality of ethnic ready-to-eat foods as a source of nourishment and food supplies chain to the remote areas become particularly important. Consuming healthy and balanced nutritional foods means eating better quality foods in proper quantities. Such foods can be developed through a preservation technique by using ionizing radiation. Although implementation of the technology for certain foodstuffs has been implemented in some countries, application of the process to a complete set of meals for selected target groups is still very limited. The various recipes of ready-to-eat food rations based on soy bean, fish, red meat, and poultry, were first developed in collaboration with medium-sized food enterprises prior to quality assessments. The products were individually packed and sealed either in a laminate pouch of Nylon-PE or polyester-aluminum foil-LLDPE and exposed to ionizing radiation at 8 kGy or 45 kGy, respectively, under cryogenic conditions throughout the process, to protect the essential dietary nutrients against free radical attack, and to reduce the undesirable chemical migration from packing material to the food and oxidative changes within the food matrix containing fats. The irradiated foods were stored at room temperature without impairing the overall quality. The high quality of irradiated ethnic foods, i.e., *bacem* tofu, *pepes* gold fish, *rendang* beef, *semur* beef, and *semur* chicken, have been administered through an intervention study on adult groups as landslide victims in Cikadu, Pemalang for 30 days continuously at breakfast time: 7.00–9.00 A.M. The results showed that body mass index (BMI) (kg/m^2^), skin fold caliper (SFC) (mm), hemoglobin (g/dL), and total lymphocyte counts (%) of the targeted respondents did not tend to increase (at *p* ≥ 0.05) after consuming the irradiated foods, while the albumin content (g/dL) showed a significant increase in blood serum (at *p* ≤ 0.05). Sensory attributes, such as general appearance, texture, color, taste, and odor of such foods showed good evaluation by the respondents in order to collect more information regarding local culture and eating habits, as well as the general opinion about the irradiated foods. The irradiated ethnic ready-to-eat foods were generally well accepted by the respondents, though the cost-benefit of mass production were still of great concern.

## 1. Introduction

For mostly healthy people, foodborne illness is not life-threatening. People living in remote areas, such as natural disaster victims, with lack of nutrition may have a greater risk for developing life-threatening complications from a foodborne illness. This is mostly due to lack food distribution access to reach the area. One of the effective preservation techniques in terms of food safety and quality is food irradiation. It is well known that irradiation combats pathogenic bacteria, insects, and parasites that contaminate food or cause spoilage and deterioration [1,2,3]. Irradiation, as a non-thermal process, can drastically reduce the presence of these disease-causing agents, providing a much broader margin of safety [4]. Used in combination with other food safety measures, it can drastically reduce the risk of illness for consumers [5].

Conventional methods applied to sterilize food include, primarily, autoclaving, microwaving, and ultraviolet irradiation, but introduces some adverse effects. Other thermal sterilization methods in order to prepare a clean diet can be accepted, though some detrimental effects on certain quality parameters still occur [6]. Ready-to-eat (RTE) foods can be pasteurized or sterilized by irradiation in the final package and can then be reheated by microwave cooking prior to serving, and some insignificant nutritional losses may occur. Nevertheless, loss of micro- and macro-nutrients can be suppressed by selecting the appropriate irradiation conditions, and proper packaging material used for this purpose.

In intensive in vitro and in vivo studies as a part of a risk assessment monitoring program, a high irradiation dose at 45 kGy of irradiated ethnic RTE showed promising results that such foods are safe and wholesome [7,8,9]. Due to some benefits of the process, high-dose irradiation can also potentially facilitate the availability of food for emergency rations as a source of nourishment and solace with a variability in menu, in which the food can be kept for long periods of time [10,11]. However, the particular foodstuffs, in these meal combinations, examined in this manuscript have not been investigated. Prevention of malnutrition is one of the keys to extend the lifespan of people who suffer from natural disasters who live in remote areas due to lack of appetite. The prevention effort is also aimed to protect them from further opportunistic infection. There are many ways to deal with these problems and improve their quality of life. 

The objective of the research was to investigate the potential benefit of producing irradiated ethnic RTE foods suitable for emergency rations, to gain ethical consent of landslide victims in a remote area, and to develop a cost-benefit approach to reduce dependency on cooling units during distribution with stakeholders in Indonesia.

## 2. Materials and Methods 

### 2.1. Materials 

Development of some other ethnic foods, such as *bacem* tofu, were selected as the main meals at breakfast time, derived from plant origin, can probably be eaten by a vegetarian group, if any. The samples were purchased from the processed tofu industry in Bogor and irradiated at a medium dose of 8 kGy at low temperature (0–3 °C) throughout the process. Other separate samples, i.e., radiation sterilization of RTE foods based on three types of animal origins, including fish base (*pepes* gold fish), meat base (*rendang* beef and *semur* beef), and poultry base (*semur* chicken), were also prepared by home industry as a medium enterprise, in order to study the effects of sterile foods on nutritional status of the respondents. Dry ice as purchased from a dry ice-making company in Jakarta, and the selected packaging materials, nylon/PE, for processed tofu, and polyester/Al-foil/LLDPE, for sterile foods, were used. Styrofoam boxes with dimensions of l × w × h = 51.25 × 36.25 × 33.75 cm was used to keep the products during and after irradiation. 

### 2.2. Methods

The preparation of making ethnic ready-to-eat foods were done by food enterprises. The radiation sterilization at 45 kGy of ethnic ready-to eat-foods basically made from fish and red meat origins, respectively, were prepared according to standard operating procedures as provided by an accredited (National Accreditation Committee for R and D/KNAPPP) food irradiation laboratory in BATAN, Jakarta, which was also applied to the previous work [12,13]. Irradiation treatment was done with an IRKA cobalt-60 irradiator, CIRA (Center for Isotopes and Radiation Application) Jakarta, Indonesia at a dose rate of 5 kGy/h. Ready-to-eat tofu was individually packed in nylon-PE, then irradiated at 8 kGy under cryogenic conditions using dry ice [14,15,16]. Products were kept under cryogenic conditions during the irradiation process, then removed to room temperature after thawing. Non-irradiated ethnic RTE foods as a control sample were also performed during the work. 

This research work has been conducted in the camp area of landslide disaster victims who stayed in the Public Health Centre building, Cikadu, Central Java for about three months after the disaster occurred. Both the non-irradiated and irradiated ethnic ready-to-eat foods were delivered over a distance of thousands of kilometers, carried from Jakarta (western part of Java) to Cikadu village in Pemalang district (Central Java). The non-irradiated foods were kept in a Styrofoam box filled with normal ice blocks during transportation. As soon as the products arrived, the non-irradiated foods were directly moved into a refrigerator to maintain the quality; meanwhile, the irradiated foods were simply kept in a plastic container at room temperature. 

**Figure 1:**
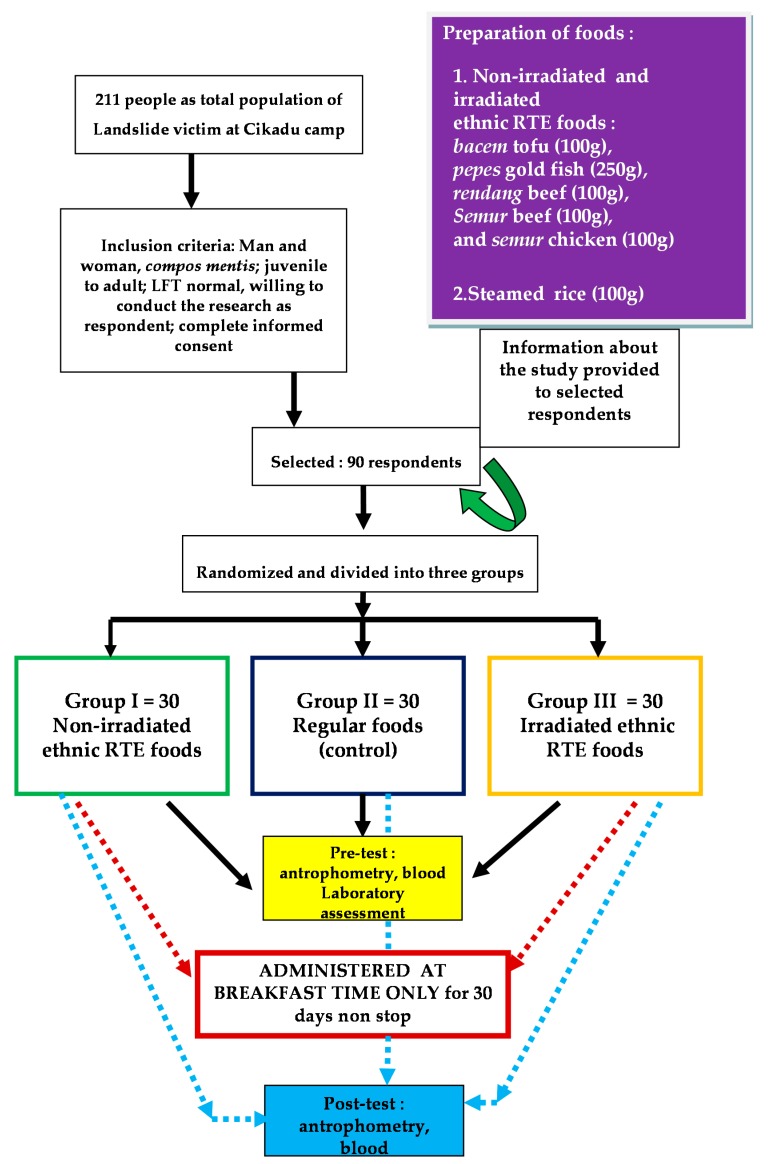
Flowchart of pre and post tests during intervention activities at Cikadu camp.

Pre and post tests during intervention studies were conducted according to Figure 1. 

Some physical parameters, as well as anthropometry, such as body mass index (BMI), including body weight and height, were measured using a calibrated weighing scale and microtoise, respectively. A skin fold caliper (SFC) was used as a tool to estimate body fat mass (BFM) to monitor the effect of diet on muscle tissue and fat. The measurement was applied at two positions on the biceps [17]. This measurement may indicate nutritional status and determine the potential risk of degenerative disease of the respondents after a natural disaster.

Blood was collected by an accredited clinical laboratory in Tegal, Central Java, for further analysis using the following parameters: hemoglobin, albumin (spectrophotometry), and total lymphocyte counts (laser light scattering).

Intervention studies of radiation sterilization RTE foods on landslide victim respondents was done at breakfast time, 7–9.00 A.M. for 30 days, continuously. The selected respondents, 90 people, fulfilled the inclusion criteria, were then randomized and split into three groups. Each group member consisted of 30 respondents, and received three different types of food. The groups also received some information and guidance regarding the purpose of the intervention study and nutrition education prior to testing [18,19]. Group I was treated with some non-irradiated foods (0 kGy) prepared by BATAN, Group II was considered as the control, with respondents getting their own breakfast and prepared from a conventional kitchen, and Group III was treated with some main meals of irradiated foods (45 kGy) prepared by BATAN.

The cycles of five-day menus, for 30 day duration, of intervention studies on ethnic ready-to-eat foods for respondents in Cikadu camp, Pemalang is presented in Table 1. Rice was served fresh immediately after cooking. Respondents should eat the meals according to the schedule and timeline. Children did not join the test some extra snacks and breads within 30 days were distributed too, while their parents consumed meals during the intervention period. Sensory evaluations of *bacem* tofu, *pepes* gold fish, *rendang* beef, *semur* beef, and *semur* chicken were conducted by the respondents in Group I (RTE foods at 0 kGy) and III (RTE foods at 45 kGy), respectively during intervention at breakfast time.

### 2.3. Quality Assessments

The effect of the intervention study of RTE foods on each respondent was evaluated. The assessment was taken both at pre and post test, according to different parameters; namely BMI (kg/m^2^), biceps (mm), blood serum, such as albumin (g/dL), hemoglobin (g/dL), and total lymphocyte count (%). In this study, the albumin parameter was considered as the best indicator to evaluate the improvement of nutritional status of respondents. This is because albumin can be synthesized within 21 days after intervention.

All collected data, including laboratory assessments, were analyzed using statistical software (SPSS 16.0, IBM, New York, NY, USA) and tested for its significance. 

The quality of the individual type of ethnic RTE foods was evaluated based on subjective measures represented as sensory evaluation, i.e., general appearance, texture, flavor, taste, and odor, according to a hedonic scale with five degrees of preference. The assessments were conducted by all respondents. For two different groups, Group I and Group III, respectively, informal interviews were also conducted to every respondent in order to get a general idea of the irradiated product acceptability.

## 3. Results

Results of anthropometry and blood serum measurements both were taken at pre and post tests are presented in Table 2. It shows that BMI (kg/m^2^) and biceps values of all groups were mostly stable before and after the tests and did not show any significant increase after intervention (at *p* ≥ 0.05). Albumin content (g/dL) of blood serum respondents of Group III (4.45) showed a significant increase by ANOVA (SPSS 16.0) at *p* ≤ 0.05 after consuming irradiated RTE foods compared to Group I (4.23) and Group II (4.26). Hemoglobin content (g/dL), as well as total lymphocyte count (%), did not show any significant decrease (at *p* ≥ 0.05) after intervention treatment (Group III: 32.83%) compared to Group I (34.40 %) and II (34.01%), respectively.

**Figure 2:**
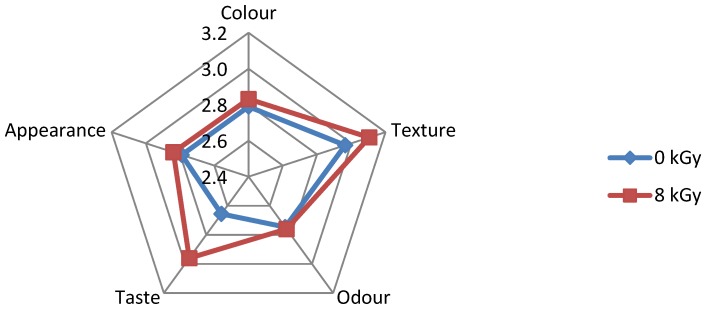
Sensory evaluation of non-irradiated and irradiated *bacem* tofu.

**Figure 3:**
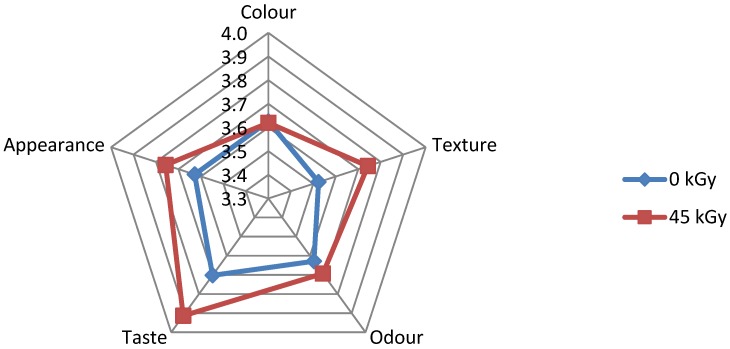
Sensory evaluation of non-irradiated and irradiated *pepes* gold fish.

**Figure 4:**
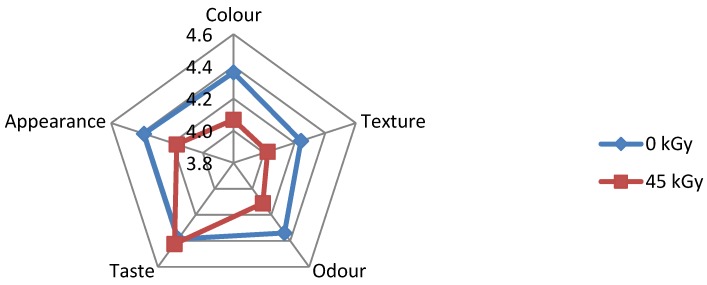
Sensory evaluation of non-irradiated and irradiated *rendang* beef.

**Figure 5:**
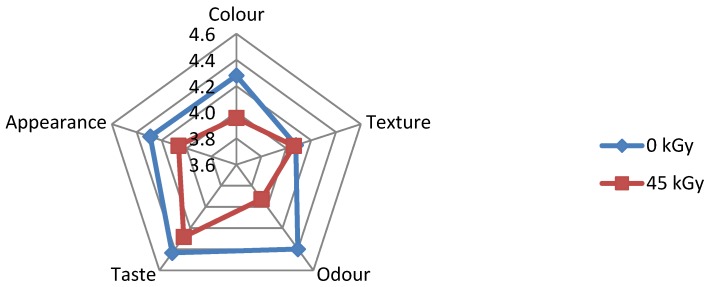
Sensory evaluation of non-irradiated and irradiated *semur* beef.

**Figure 6:**
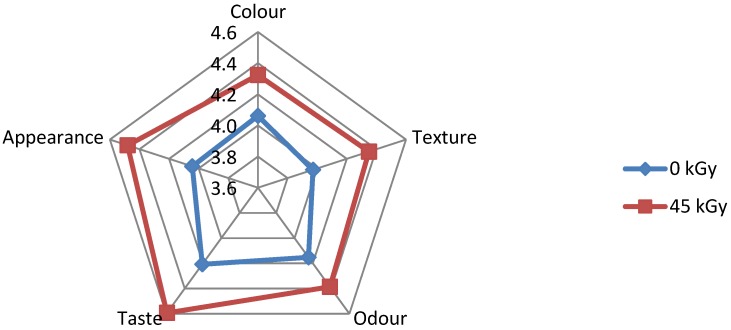
Sensory evaluation of non-irradiated and irradiated *semur* chicken.

The results of sensory evaluation of irradiated and non-irradiated foods, as performed by Group I and Group III, respectively, are illustrated in Figure 2, Figure 3, Figure 4, Figure 5 and Figure 6. It is shown that some respondents in each group like all types of ethnic ready-to-eat foods prepared by BATAN. According to the questionnaire distributed among volunteer respondents, irradiated *semur* chicken was the most favorite meal, followed by irradiated *pepes* gold fish, rather than the other irradiated products. Figure 2 shows that data on the sensory evaluation of irradiated tofu both at a dose of 8 kGy and non-irradiated one remained stable. Similar results were also obtained for the appearance, color, and odor.

Figure 3 shows that texture and taste of irradiated *pepes* gold fish at sterilization dose, 45 kGy, gave better results than the non-irradiated one, while color and odor show similar results.

It is obvious from Figure 4 and Figure 6, respectively that irradiated ready to eat foods prepared from red meat base did not show any good values in sensory evaluation.

## 4. Discussion

The overall result showed that BMI from Group I, II, and III were still in the range of normal limits (18–25 kg/m^2^) both at pre and post tests. There was also positive correlation between BMI and BFM obtained at post test. This result could figure out that nutrition status of the respondents were still normal, anthropometrically. Albumin level in the blood is the most important indicator of health status. Albumin serum is also one important parameter to indicate the degree of morbidity and mortality in the human body. Increasing albumin content in the blood serum of group III revealed that different types of protein compounds in irradiated foods could be better digested than the non-irradiated one.

It is well known that ionizing radiation imparts an effect on the food matrix and leads to degradation rather than cross-linking. The degradation might soften the tissue and make it easy to digest. This issue could probably be stated as scientific evidence that slow release of some nutritive food constituents which are trapped in the matrix might take place. Intensive studies on some irradiated ethnic RTE foods in vitro [7,8], animal feeding study in vivo [9], and food spectra using nanotechnology [20,21] show that antioxidant capacity tends to increase statistically, and could probably confirm the evidence. High quality, hygienic, and wholesome irradiated ethnic ready-to-eat foods could give a positive contribution [22] to improving the quality of life of the respondents. Some researchers reported that reducing body mass cells and albumin serum might increase the morbidity [19].

Lymphocytes play a major role in increasing body immunity. Separate studies on HIV patients in China [23] concluded that, when considering the antiretroviral therapy for HIV-infected Chinese individuals, total lymphocyte count can be considered as an inexpensive and easily available surrogate marker for predicting two clinically-important thresholds of CD4 count of 350 cells/mm^3^ and 500 cells/mm^3^.

The intervention study in this study, as well as those reported in previous works [24,25], resulted in total lymphocyte counts of the respondents being calculated based on total leucocyte counts. The stability of lymphocyte counts after the test may reflect the immune system of respondents were still compromised.

All of the respondents of Group I, Group II, and Group III were very cooperative to conduct the test. An interesting information based on their eating habit of the respondents could be withdrawn. The taste of *Rendang* beef was probably a bit spicy since the respondents are not familiar with such products. It should be noted that different tribes might have different typical local foods which are most favorable. *Rendang* meat is considered as a strange taste for the local respondent. Based on that experience, lessons were learned that providing foods as emergency rations can only be well-consumed by the people as long as we consider the most favorable taste and general appearance for them.

The respondents were used to preparing and consuming ready-to-eat foods based on beans, fish, and chicken, rather than red meat. Fish and chicken could be easily provided from their own farms before the natural disaster struck their village. 

## 5. Techno-Economy Feasibility

A simple techno-economical approach was estimated in order to calculate the irradiation cost of ethnic RTE foods. The irradiation cost of ethnic RTE foods at the dose of 8 kGy is about USD 0.08/100 g product, and at the dose of 45 kGy is about USD 0.43/100 g product, respectively.

### 5.1. Cost Benefit Approach to Irradiate Ethnic Ready-to-Eat Foods in a Commercial Irradiator

It is assumed that medium food enterprises are going to produce the irradiated foods at a Category IV commercial irradiation facility.

There is an approach to calculate the irradiation cost at irradiation facilities to conduct the service. Estimation tax rate (within one week): 1 USD = Rp. 12,000 (twelve thousand Rupiah).

a. Irradiation of ethnic RTE foods at a medium dose (8–10 kGy)

Specification: Radiation dose required: min. 8 kGy; target cycle time: 9 min; radiation time: 13.5 h; load capacity: 180 kg/tote, mantel tote dimension: 86 × 56 × 116 cm^3^; price of dry ice/kg: Rp. 11,000. Total dry ice needed: 17 kg/tote; total price for dry ice: Rp. 204,000/tote. Total price of irradiation service with dry ice: Rp. 1,536,000/tote + 10% tax + stamp: Rp. 1,695,600; radiation service + dry ice/kg product: Rp. 9420 or radiation service + dry ice/100 g product: Rp.942 or USD 0.08/100 g product.

b. Irradiation of ethnic RTE foods at high dose (Min. 45 kGy)

Specification: radiation dose required: min. 45 kGy; target cycle time: 50 min; radiation time: 75 h; load capacity: 180 kg/tote, mantel tote dimension: 86 × 56 × 116 cm^3^; price of dry ice/kg: Rp. 11,000; total dry ice needed: 94 kg/tote; total price for dry ice: Rp. 1,034,000; total price of irradiation service with dry ice: Rp. 8,402,000 + 10% Tax + stamp: Rp. 9,248,200; radiation service + dry ice/kg product: Rp. 51,379 or radiation service + dry ice/100 g product: Rp. 5138 or USD 0.43/100 g product.

## 6. Conclusions and Recommendation

It can be concluded from this study that irradiated ethnic RTE foods derived from plant origins at a dose of 8 kGy, and derived from animal origin at a dose of 45 kGy, could be introduced to people who suffer from natural disasters to improve their nutritional status. It can be suggested that type and composition of diets should be, first, well-defined, and then adjusted according to the favorable dishes of the targeted people before conducting the test. The local people who joined the group as respondents should be very keen and cooperative during the observation and interview. The irradiation cost of ethnic ready-to-eat foods both at the dose of 8 kGy and of 45 kGy are still reasonable.

## Figures and Tables

**Table 1:** Cyclus of 5 day menu for 30 days duration of intervention studies on ethnic ready to eat foods for respondents in Cikadu camp. The type of menus were repeated and administered to the targeted respondents on 5 day basis.

Day	Menu
1st 2nd 3rd 4th 5th	Rice and *rendang* beef Rice and *bacem* tofu Rice and *semur* beef Rice and *pepes* gold fish Rice and *semur* chicken

**Table 2:** Results of anthropometry and blood test of respondents before (pre-test) and after intervention ethnic ready-to-eat foods (post-test) of three groups.

Parameter	Group I * (*n* = 30)	Group II ** (*n* = 30)	Group III *** (*n* = 30)
BMI (kg/m^2^) pre test	23.22 ± 3.82	23.13 ± 3.94	23.03 ± 4.39
BMI (kg/m^2^) post test	23.33 ± 3.90	23.03 ± 3.88	22.96 ± 4.36
Biceps (mm) pre test	1.86 ± 0.58	2.37 ± 0.82	2.73 ± 1.20
Biceps (mm) post test	3.94 ± 1.71	3.59 ± 1.38	4.04 ± 2.18
Albumin (g/dL) pre test	4.26 ± 0.32	4.43 ± 0.30	4.37 ± 0.23
Albumin (g/dL) post test	4.23 ± 0.32	4.26 ± 0.32	4.45 ± 0.32
Hemoglobin (g/dL) pre test	13.66 ± 1.82	14.03 ± 2.22	14.38 ± 1.25
Hemoglobin (g/dL) post test	13.35 ± 1.88	13.67 ± 2.08	14.14 ± 1.33
Total lymphocyte counts (%) pre test	34.00 ± 6.96	33.50 ± 7.5	33.58 ± 7.29
Total lymphocyte counts (%) post test	34.40 ± 7.89	34.01 ± 8.00	32.83 ± 8.40

Note: * respondents consumed non-irradiated, ethnic ready-to-eat foods; ** respondents consumed ordinary foods (control); *** respondents consumed irradiated, ethnic ready-to-eat foods.
